# The cumulative analgesic effect of repeated electroacupuncture is modulated by Adora3 in the SCDH of mice with neuropathic pain

**DOI:** 10.1002/ame2.12458

**Published:** 2024-07-11

**Authors:** Faisal Ayub Kiani, Hao Li, Panpan Guo, Qiulin Zhang, Mahmoud M. Abouelfetouh, Mingxing Ding, Yi Ding

**Affiliations:** ^1^ Clinical Veterinary Medicine, College of Veterinary Medicine Huazhong Agricultural University Wuhan China; ^2^ Department of Clinical Sciences, Faculty of Veterinary Sciences Bahauddin Zakariyah University Multan Pakistan; ^3^ Department of Surgery, Anesthesiology, and Radiology, Faculty of Veterinary Medicine Benha University Moshtohor Egypt

**Keywords:** Adora3, antinociception, electroacupuncture, lumbar spinal cord, neuropathic pain

## Abstract

**Background:**

Existing remedial approaches for relieving neuropathic pain (NPP) are challenging and open the way for alternative therapeutic measures such as electroacupuncture (EA). The mechanism underlying the antinociceptive effects of repeated EA sessions, particularly concerning the regulation of the Adora3 receptor and its associated enzymes, has remained elusive.

**Methods:**

This study used a mouse model of spared nerve injury (SNI) to explore the cumulative analgesic effects of repeated EA at ST36 (Zusanli) and its impact on Adora3 regulation in the spinal cord dorsal horn (SCDH). Forty‐eight male mice underwent SNI surgery for induction of neuropathic pain and were randomly assigned to the SNI, SNI + 2EA, SNI + 4EA, and SNI + 7EA groups. Spinal cord (L4–L6) was sampled for immunofluorescence, adenosine (ADO) detection and for molecular investigations following repeated EA treatment.

**Results:**

Following spared nerve injury (SNI), there was a significant decrease in mechanical withdrawal thresholds (PWTs) and thermal nociceptive withdrawal latency (TWL) in the ipsilateral hind paw on the third day post‐surgery, while the contralateral hind paw PWTs showed no significant changes. On subsequent EA treatments, the SNI + EA groups led to a significant increase in pain thresholds (*p* < 0.05). Repeated EA sessions in SNI mice upregulated Adenosine A3 (Adora3) and cluster of differentiation‐73 (CD73) expression while downregulating adenosine deaminase (ADA) and enhancing neuronal instigation in the SCDH. Colocalization analysis of Neun‐treated cells revealed increased Adora3 expression, particularly in the SNI + 7EA group.

**Conclusions:**

In conclusion, cumulative electroacupuncture treatment reduced neuropathic pain by regulating Adora3 and CD73 expression, inhibiting ADA and most likely increasing neuronal activation in the SCDH. This study offers a promising therapeutic option for managing neuropathic pain, paving the way for further research.

## Graphical abstract

Neuropathic pain management via repeated electroacupuncture.

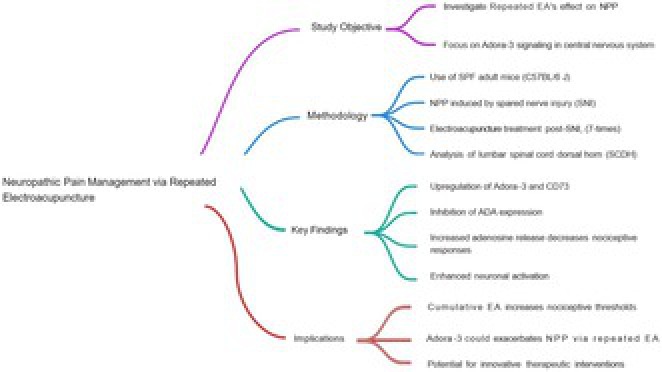

## 1 INTRODUCTION

Neuropathic pain (NPP) is an extremely painful condition caused by either injury of the nervous system or its malfunction. It presents a huge obstacle to effective treatment choices.^1^ The onset of neuropathic pain occurs with abnormal signaling within the nervous system and manifests as searing, shooting, tingling, or electric shock‐like sensations. This condition significantly impacts the quality of life of sufferers. Patients describe this painful condition as persistent, severe, or challenging to manage.^2^ The global prevalence of NPP conditions like postherpetic neuralgia, chemotherapy‐induced neuropathy, nerve injury, and diabetic neuropathy underscores its substantial societal ramifications.^3^

Acupuncture is a traditional Chinese medicine treatment option for chronic pain management. This ancient method entails the careful insertion of small needles into certain spots on the body, believed to reinstate the flow of Qi, an energy force thought to travel through specific channels or meridians.^4^, ^5^ Repeated acupuncture sessions have been efficacious in addressing chronic pain issues.^6^ Acupuncture therapy can benefit patients with osteoarthritis or chronic neck, back, or shoulder pain by reducing pain interference, depression, and severity. Research has indicated that for acupuncture to significantly reduce pain intensity and prevent the progression of chronic pain, it must be administered continuously for a minimum period of 2 weeks.^7^ This discovery could have a significant impact on the ability of those with low means to access effective acupuncture therapy.^6^ Electroacupuncture (EA), a modern variant that incorporates electrical stimulation, has gained favor in both traditional Chinese and Western medicine for its potential to ease pain, reduce inflammation, and improve general well‐being.^8^

Conventional pharmacological treatments often provide poor relief due to the complicated pathophysiology of neuropathic pain, which is characterized by maladaptive alterations in the nerve system that perpetuate the pain perception. This constraint has prompted the investigation of alternate therapies, with EA appearing as a viable option. Although its effect is established in a variety of pain situations, the exact role of repeated EA in NPP management has remained an ongoing topic of investigation. To fill this study gap, this research elucidates the cumulative analgesic effects of repeated EA in a murine model of NPP. This study specifically investigated the involvement of the Adora3 within the SCDH of experimental animals. Adenosine, a purine nucleoside, is known to play a crucial role in various physiological processes, including the regulation of neurotransmission.^9^, ^10^ Adora3, which is expressed in CNS, has been linked to modifying nociceptive signaling, making it a prospective target for pain management.^11^, ^12^ In view of this potential, the complex relationships between recurrent EA, and adenosine signaling through Adora3 in the setting of neuropathic pain require additional investigation.

Our study takes a multidisciplinary approach that includes behavioral, neurochemical, and molecular investigations, and aims to provide valuable insights into the processes underlying the cumulative analgesic effects of repeated EA. Understanding the intricate interplay between recurrent EA and Adora3 is an important area of research for developing targeted therapies for managing neuropathic pain. Our study aimed to shed light on this complex pathway, which could improve our understanding of pain control mechanisms and lead to the development of new treatment strategies that combine EA and Adora3 regulation.

## 2 METHODS

### 2.1 Experimental animals

In total, 48 male C57BL/6 mice aged 2 months, weighing between 20 and 30 g, were employed in this investigation. The mice were accommodated in groups, with no more than five mice per cage, in a climate‐controlled temperature and humidity with a 12‐h light/dark cycle. Before the experimental procedure began, the mice were given a week to acclimatize and they were fed an extruded rodent diet (Tek lad Global) with ad libitum fresh water supplied throughout the day. Ethical approval was granted (HZAU‐20240105001) for the use and care of experimental animals from the Institutional Animal Care and Use Committee of Huazhong Agricultural University by observing the regulations for the administration of affairs concerning experimental animals of China.

**FIGURE 1:**
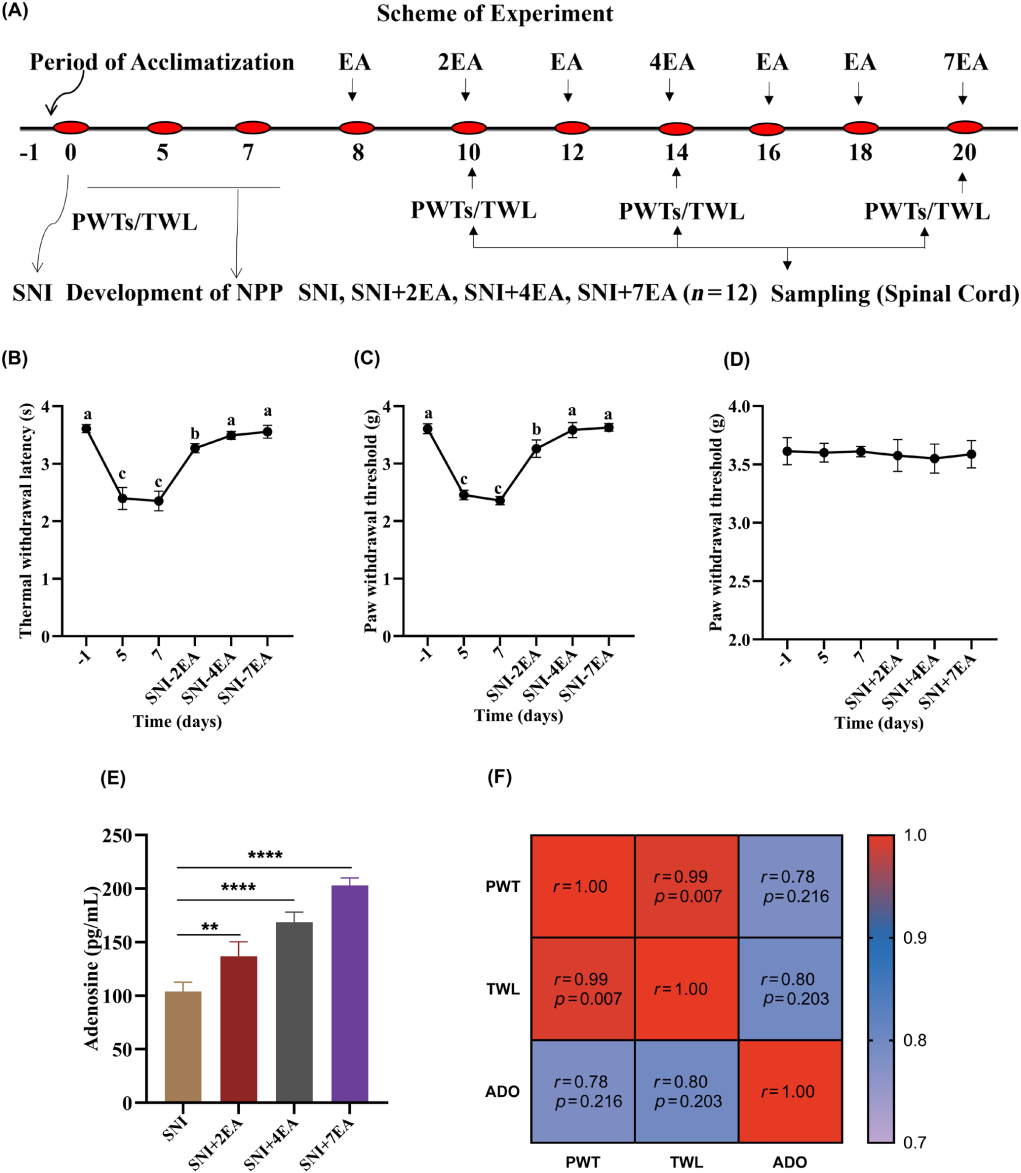
Effect of repeated EA on mechanical allodynia and thermal hyperalgesia and ADO concentration. (A) Experimental schematic diagram. (B, C) Paw withdrawal thresholds (PWTs, B) and thermal withdrawal latencies (C). (D) No change was observed in the contralateral PWT from Day 1 to Day 7 of EA. (E) Adenosine levels in the spinal cord (adenosine pg/mL). (F) Pearson's correlation analysis between ADO concentration and the nociceptive responses. The data are presented as the means ± SD. Statistical significance was considered at *p* < 0.05 (significant, **), and *p* < 0.0001 (highly significant, ****) versus SNI group. Statistical significance for pain thresholds has been shown with letters (a, b, and c) on same day (*p* < 0.05); One‐way ANOVA followed by Bonferroni correction. ADO, Adenosine; EA, electroacupuncture.

To investigate the impact of repeated electroacupuncture on the adenosine A3 receptor and its metabolizing enzymes involved in the mitigation of NPP produced by SNI, the mice were randomly assigned to four groups: SNI, SNI + 2EA, SNI + 4EA, and SNI+ 7EA (n = 12). In this study, Adora3 and ADO‐metabolizing enzymes (CD73 and ADA) were assessed through RT‐qPCR and western blotting after establishing the SNI model (Day 8) and subsequently after the 2nd EA, 4th EA and 7th EA treatments post‐surgery (Figure 1A). Adenosine metabolism is regulated by both 5′‐nucleotidase (CD73) and adenosine deaminase (ADA) enzymes.^13^, ^14^ Mechanical and thermal sensitivities were recorded three times before and on days 2EA, 4EA, and 7EA of SNI surgery following the first EA session. After measuring nociceptive thresholds on respective repeated EA sessions, the spinal cord samples were collected for immunofluorescence staining and molecular investigations.

### 2.2 Surgical intervention for spared nerve injury

The mice were fasted for 24 h before the surgical intervention for SNI modeling. Surgical intervention for spared nerve injury (SNI) was performed using established protocols to induce NPP.^15^ For the surgical procedure, the mice were anesthetized with 1% sodium pentobarbital for the surgery.^16^ The skin at the surgical location on the left hind limb was exposed by shaving and alcohol swabs and iodine were used to sterilize the skin. Using a surgical scalpel, an incision 0.5–1 cm long was made above the midpoint of the line connecting the head of the tibia to the greater trochanter of the femur. The sciatic nerve trunk and branches were later made visible by abrupt dissection, which also exposed the underlying muscles and tissues. Non‐absorbent (6/0) sutures were used to ligate the peroneal and sural branches firmly, and the ligatures were transected below. After that, the tibial nerve was left intact and a 2‐mm portion of the nerve stump distal to the ligature was carefully excised. Layer by layer, the incision was sutured and the surgical site was cleaned. The entire process was carried out in a sterile environment. The mouse groups underwent multiple EA sessions at the designated time after the NPP was established 7 days following SNI surgery (Figure 1A).

### 2.3 EA stimulation

EA treatment was administered seven times at 8, 10, 12, 14, 16, 18, and 20 days post‐SNI surgery (Figure 1A). EA stimulation was initiated on Day 8 after SNI surgery, and the mice were adequately confined in a specially built contraption that exposed their legs. In each of the mice's hind limbs, an acupoint ST36 was positioned 3–4 mm below and 1–2 mm across the knee midline, with a sterile acupuncture needle (0.30 × 5 mm; Zhongyan Taihe Medi. Instrument Co. Ltd, Beijing, China) inserted perpendicularly to a depth of 4 mm.^17^ Afterwards, an electrical nerve stimulation was produced using HANS‐200E (Jisheng Medi. Instruments, Nanjing, China). The mice received EA (2/100 Hz, 0.5–1.5 mA) at ST36 for 30 min on treatment days.^18^ The EA regimens used in this study ranged between low‐frequency (2 Hz) and high‐frequency (100 Hz) stimulation (known as 2/100 EA) to produce analgesic effects. The intensity/strength was based on a slight contraction of the muscle following EA stimulation, which was 0.5–1.5 mA in the present study. All the mice were alert and conscious during the whole EA stimulation procedure.

### 2.4 Mechanical withdrawal test (PWT)

In this project, an electronic von Frey anesthesiometer (16 001, IITC Life Science, Woodland Hills, CA, USA) was utilized to observe mechanical allodynia (paw withdrawal thresholds) 3 days before SNI and after the 2nd, 4th and 7th EA sessions, as depicted in Figure 1A. The nociceptive pain threshold in each mouse was determined by the mechanical stimulation at the plantar surface that corresponded to the withdraw of the hind paw.^19^ Briefly, SNI mice were placed one at a time across a wire mesh ledge in a bottomless cage (measuring: 20 × 15 × 15 cm) including many compartments. A punctuate stimulus was applied below the wire mesh floor to the mouse's hind paw through the even tip of the von Frey filament, and then pressure was applied (the range of von Frey filament forces spanned from 0.07 to 1.4 g). If the animal withdrew the paw, a response was automatically read by the von Frey instrument. Each hind paw was tested five times at 5‐min intervals, with the average value recorded.

### 2.5 Thermal withdrawal latency (TWL)

Utilizing a hot plantar test device (BIO‐CHP, Bioseb, France) thermal hyperalgesia was assessed. Each SNI mouse was placed at a consistent temperature of 52.5 ± 1°C on a hot plate. An electronic timer was used to record the amount of time needed for reactions, like jumping or licking the hind paw, after the animals had been acclimated for 15 min.^20^ To avoid blistering or scorching of the paws, the maximum heating duration was set to 20 s. Each mouse was treated to a maximum of three TWL tests at intervals of at least 5 min. Normal paw‐lifting motions were not considered to reduce bias.

### 2.6 Sample collection

The mice were euthanized to collect the spinal cord (L4–L6) for investigating the impact of repeated electroacupuncture (EA) on neuropathic pain management and the expression levels of the Adora‐3 Receptor. Following the respective EA treatment sessions, three samples per group were preserved in formalin for immunofluorescence analysis, while another three per group were immediately processed to determine spinal ADO concentration using an ELISA ADO Kit following the manufacturer's instructions. The spinal cords from remaining six mice in each group were promptly frozen in liquid nitrogen and stored at −80°C for subsequent western blotting and RT‐PCR analysis.

### 2.7 Enzyme‐linked immunosorbent assays (ELISAs)

After the respective EA sessions, the spinal cord samples were immediately placed into dry ice and then processed for determination of adenosine concentration. To measure ADO levels in spinal cord samples, an ELISA ADO kit (JM‐11538M1, Jiangsu Jingmei Biological Technology Co., Ltd, Yancheng, China) was used. The whole protocol described by the manufacturer was strictly followed to measure the adenosine levels.

### 2.8 RNA separation and real‐time quantitative PCR

The spinal cord samples were kept in liquid nitrogen and crushed into powder. Total RNA was extracted from the samples using TRIzol (CW0581, CWbio Co. Ltd, Beijing, China). After normalizing the extracted pure total RNA concentrations, the Superscript II First‐Strand Synthesis System (AB‐Clonal Biotechnology, Wuhan, China) was used to reverse transcribe the RNA into cDNA. To prepare cDNA, the RNA was heated at 65°C for 5 min along with random hexamer primers and dNTP mixture. Then, cDNA synthesis buffer and dithiothreitol were added, followed by reverse transcriptase to create first‐strand cDNA. A dilution of the cDNA was kept at −80°C until the RT‐qPCR analysis. The primer sequences utilized were sourced from GenBank at the National Centre for Biotechnology Information (NCBI) (Table 1). Using a MyiQ Single‐Color Real‐Time PCR Detection System (Bio‐Rad) and 96‐well PCR plates, cDNA amplification was carried out using the Quanti Tect SYBR Green PCR Kit (QIAGEN). The reaction mixture for amplification included 2 μL of cDNA, 10 μL of REAL SYBR mixture (2×), 0.8 μL (10 μmol/μL) of forward and reverse primers, and 7.2 μL of PCR‐grade water, for a total volume of 20 μL. PCR was carried out under the following conditions: 10 min at 95°C (pre‐denaturation), 40 cycles of 15 s at 95°C (denaturation), 60 s at 60°C (annealing), and 25 s at 72°C (extension), with fluorescence acquisition following each cycle. Finally, a dissociation curve was obtained by increasing the temperature from 65 to 95°C to verify primer specificity. To prevent differences between runs, all samples for every reference gene were performed on the same plate. The relative mRNA levels are expressed as 2^−ΔΔCT^ values, and the relative gene expression was measured using the ΔΔCT method.

**TABLE 1:** The sequences of primers used for experimental procedures were as follows.

Gene	Sequence	Gene bank accession No.
Adenosine A3	F‐5 (GGAAGCCGACAACACCACG)3	NM009631.4
	R‐5 (CTTGACCACCCAGATGACCAG)3	
CD73	F‐5 (AGCGATGACTCCACCAAGIG)3	NM011851.4
	R‐5 (CAGATGGTGCCCTGGTACIG)3	
Adenosine Deaminase	F‐5 (CGACTGAACATCAACGCAGC)3	NM001272052.1
	R‐5 (TCAGTCTGTGGTGGCTATTGG)3	
GAPDH	F‐5 (TGAGCCTCCTCCAATTCAACC)3	NM0011289726.2
	R‐5 (AATCCGTTCACACCGACCTT)3	

### 2.9 Western blot analysis

Total protein was extracted from spinal cord samples by homogenizing the protein lysate with protease and phosphatase inhibitors (Roche, Shanghai, China). The protein content was measured using the bicinchoninic acid (BCA) protein assay. Protein samples were separated using 10% SDS‐PAGE and transferred to a PVDF membrane at 120 mA for 2 h. Nonfat milk (5%) dissolved in TBST was used to block the membranes for 1 h at room temperature. The membranes were then incubated for 16 h at 4°C with the following primary antibodies: A3 (Affinity Biosciences: DF4851, diluted 1:500); a polyclonal antibody CD73 (Protein tech 12 231‐1‐AP, diluted 1:1000); a polyclonal antibody ADA (Protein tech 13 328‐1‐AP, diluted 1:500); and a GAPDH antibody (AB‐Clonal, AC001; Wuhan, China; 1:3000) diluted in 5% BSA. Following washing, secondary antibodies conjugated with horseradish peroxidase were applied to the membranes for 2 h at 30°C (donkey anti‐rabbit IgG; AB‐Clonal, AS038; Wuhan, China; 1:10000 in TBST). Upon incubation, protein bands on the membranes were identified using an enhanced chemiluminescence (ECL) detection technique. Images were captured using the automatic molecular imaging system FUSION SOLO S (VILBER BIO IMAGING, Vilber, France), and the optical density of the bands was determined using ImageJ software (Wayne Rasband, National Institutes of Health, USA). The protein density of GAPDH served as an internal control.

### 2.10 Immunofluorescence labeling

Following extraction of the spinal cord (L4–L6), the samples were fixed in 4% paraformaldehyde solution for 2 h. The samples were embedded in paraffin, and each block was consecutively sectioned at a thickness of 5 μm. The paraffin slides holding spinal cord tissue were incubated for 2 h at 37°C and then dewaxed to water (Xylene I 15 min, Xylene II 15 min, Xylene II 10 min, 100% Ethanol I 3 min, 100% Ethanol II 3 min, 95% Ethanol 3 min, 80% Ethanol 3 min, and 75% Ethanol 3 min) and washed three times with PBS. Slides were then immersed in the citrate repair solution, and a steamer was used to heat the repair solution to 92–98°C. The slides were maintained at this temperature for 20 min, then naturally cooled to room temperature, and finally washed with PBS 3 times for 5 min. A 5% BSA blocking solution was added dropwise to the tissues, which were then incubated at 37°C for an hour and washed with PBS. The sections were then treated with an Adora3 polyclonal antibody (Thermo Fisher Invitrogen, diluted 1:200), an anti‐NeuN antibody (Protein tech 66 836‐1‐Ig, diluted 1:200), and an anti‐GFAP antibody (CST Protein tech 66 836‐1‐Ig, diluted 1:200) at 4°C overnight. After washing with PBS, the sections were treated with dyes Cy3 or FITC‐labeled anti‐rabbit IgG at the appropriate dilution applied dropwise to the slides, and then incubated for 30 min at 37°C before being washed with PBS. Preferential incubation for Adora3 was performed with stable red Cy3 (1:1000). The slides were then counterstained with DAPI (4,6‐diamidino‐2‐phenylindole) for 10 min at room temperature, washed, then probed with antifade mounting medium to protect them from light, and images were recorded and analyzed using ImageJ software to determine fluorescence intensity.

### 2.11 Statistical analysis

Version 8.0.1 of GraphPad Prism was used for statistical analysis. The means and standard deviations (SDs) of the quantitative data are displayed. Following data normalization, the nociceptive behaviors were analyzed by one‐way ANOVA, with different treatments used as primary variables. To compare the RT‐PCR and western blot data, one‐way ANOVA was employed. To ascertain the statistical significance of the differences between the variables (*p* value < 0.05), the Bonferroni correction was applied.

## 3 RESULTS

### 3.1 Effect of repeated EA on adenosine levels, mechanical allodynia and thermal hyperalgesia in SNI mice

In the present study, baseline nociceptive thresholds were measured in a murine model as a reference point, after which nociception and adenosine concentrations were recorded after SNI was established and after the 2nd, 4th and 7th EA sessions. Ipsilateral PWT and TWL values indicated that nociception before SNI (Day 1) was significantly higher and decreased measurably after SNI (Day 0–7), indicating the successful establishment of the NPP model. PWT and TWL values were restored to normal after multiple EA sessions (Figure 1B,C). No significant difference was observed in the pain thresholds of the contralateral hind paw during the observational period (Figure 1D). Throughout multiple EA treatments, adenosine levels exhibited an increasing trend with advancing time, suggesting that electroacupuncture had a cumulative effect on adenosine release (Figure 1E). In the SNI groups, a notable increase in adenosine levels was observed following the EA sessions, and this effect was significantly higher in SNI + 7EA group mice (*p* < 0.05). Furthermore, Pearson's correlation analysis between the nociceptive responses and adenosine concentration indicated that the adenosine concentration was significantly and positively correlated with the PWT and TWL (Figure 1F).

### 3.2 The impact of repeated EA sessions on the elevation of the spinal Adora3 receptor mRNA and protein levels in SCDH

**FIGURE 2:**
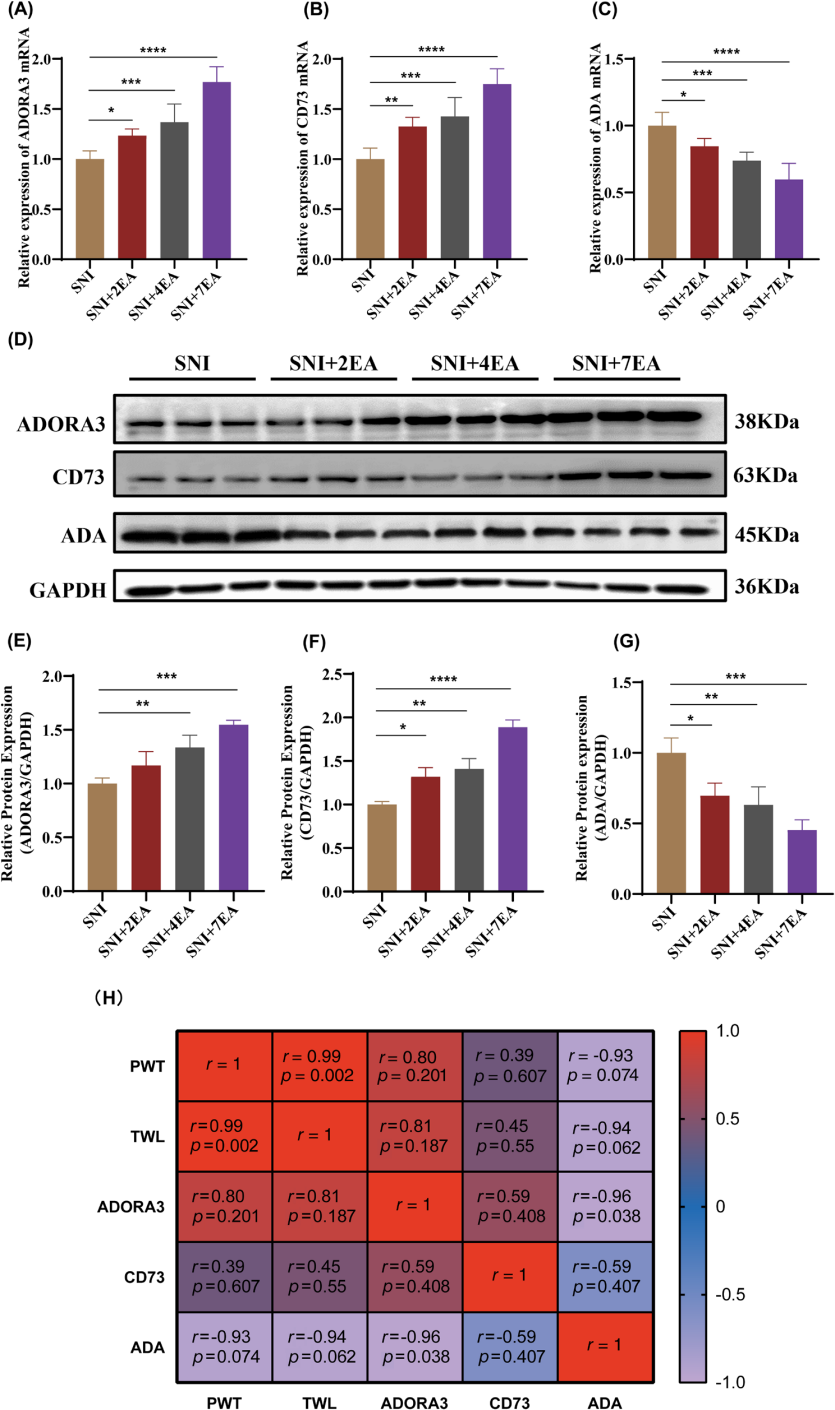
Multiple electroacupuncture sessions influence the Spinal Adora3 and related enzymes. (A–C) Relative mRNA expression levels of Adora3, CD73, and ADA. (D) Western blot showing protein expression. (E–G) Relative protein expression levels of Adora3, CD73, ADA, and GAPDH. (H) Pearson's correlation analysis of Adora3 and metabolizing enzymes with the nociceptive responses. On days 2EA, 4EA, and 7EA following surgery, samples were obtained from the lumber L4–L6 segments. The data are presented as the means ± SD. Statistical significance was considered at *p* < 0.05 (significant, *), *p* < 0.01 (**), *p* < 0.001 (***), and *p* < 0.0001 (highly significant, ****) compared to the SNI group; *n* = 6. One‐way ANOVA followed by Bonferroni correction.

The impact of multiple EA treatments on the relative mRNA and protein expressions of the spinal Adora3 receptor, adenosine metabolizing enzymes CD73 and ADA are presented in Figure 2A–G. After the 2nd, 4th and 7th EA sessions post‐SNI, a substantial increase (*p* < 0.0001) in Adora3 and CD73 mRNA expression was observed in the SCDH of the SNI + 7EA group compared to that in the SNI group (Figure 2A,B). In contrast, ADA mRNA expression was decreased in the SNI + 7EA group (Figure 2C). Furthermore, the protein expression of Adora3 and CD73 was significantly (*p* < 0.001) upregulated in the SCDH of SNI + 7EA mice compared to that in the other groups (Figure 2E,F). Conversely, SNI + 7EA mice exhibited a marked reduction in spinal ADA protein expression compared to the other groups (Figure 2G). Additionally, Pearson's correlation analysis between Adora3, CD73 and the nociceptive responses indicated a significantly positive correlation whereas, PWT and TWL indicated a negative correlation with ADA (Figure 2H).

### 3.3 The impact of repeated EA on the florescence intensity of the Adora3 receptor in SCDH of SNI mice

**FIGURE 3:**
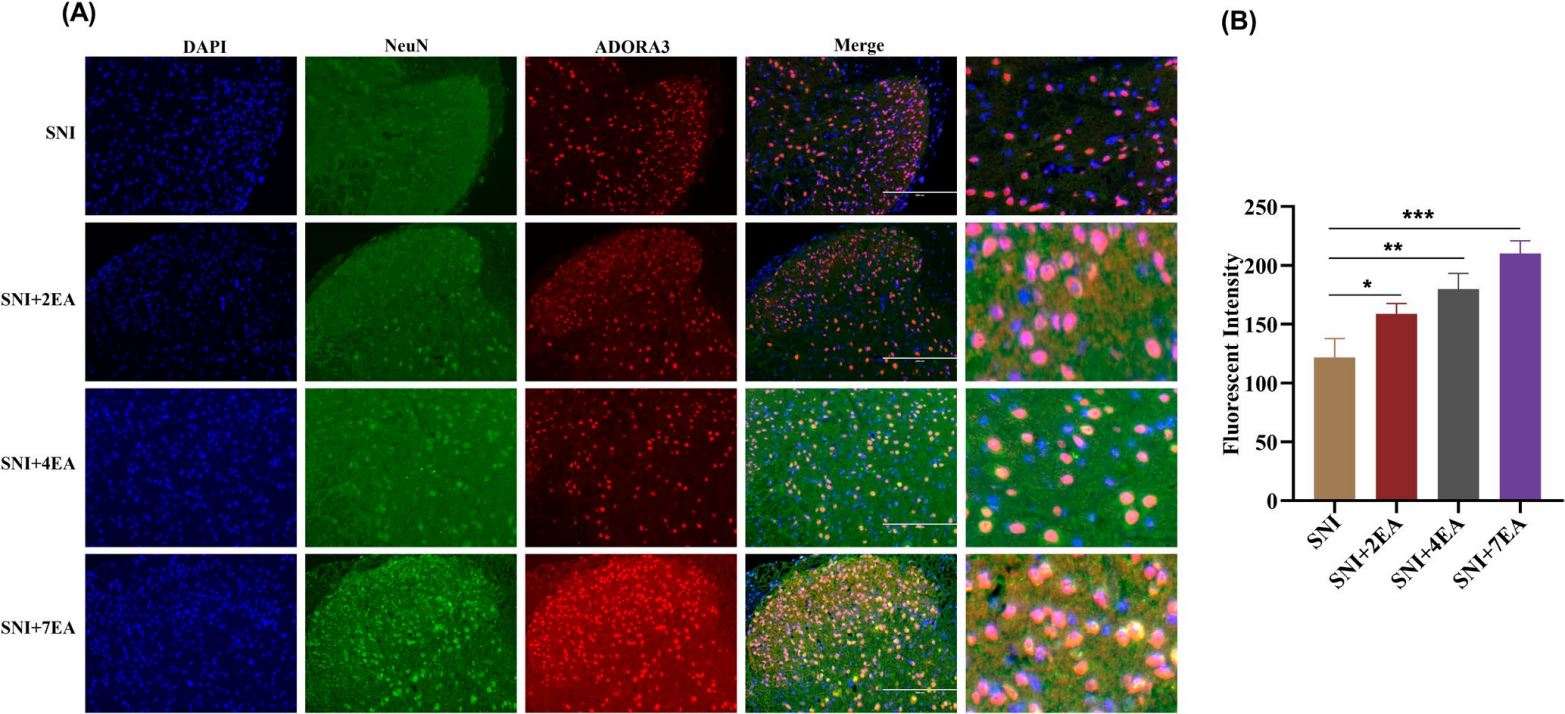
Immunofluorescence staining was used to determine the effect of repeated EA on the co‐expression of spinal Adora3 in SNI mice. (A) Adora3 receptors were double stained in the SCDH from the SNI and SNI+ EA groups (scale bar: 200 μm). (B) The mean fluorescence intensity of the Adora3 in SCDHs after cumulative electroacupuncture. The data are presented as means ± SDs. Statistical significance was considered at *p* < 0.05 (significant, *), *p* < 0.01 (**), and *p* < 0.001 (highly significant, ***) compared to the SNI group; *n* = 3. One‐way ANOVA followed by the Bonferroni correction test.

The impact of multiple EA sessions on the colocalization and expression of Adora3 adenosine receptors with neuronal cells in the SCDH is shown in (Figure 3A,B). Immunofluorescence staining was performed using the neuronal marker NeuN to assess the co‐expression of Adora3. Immunofluorescence staining showed a significant increase in the Adora3 levels after cumulative EA stimulation. The results showed a significant increase in fluorescence intensity when comparing the Adora3 levels in different treatment groups (2EA, 4EA, and 7EA, respectively) to those in the SNI group. The statistical analysis indicated high significance levels (*p* < 0.01, *p* < 0.001, and *p* < 0.0001, respectively), highlighting that the multiple EA sessions are modulated by expressions of Adora3 receptor (Figure 3B). These findings suggest a potential mechanism through which multiple sessions of EA exert antinociceptive effects, possibly by modulating adenosine signaling and attenuating neuropathic pain.

## 4 DISCUSSION

Chronic pain lasts for a long period with different conditions and intensities. Chronic pain is frequently accompanied by other health issues such as exhaustion, sleep disturbance, decreased appetite and mood fluctuations, which have a negative impact on the patient's health.^21^ Neuropathic pain is a type of pain that results from damage to or malfunctions of the nervous system and can also accompanied by chronic pain. It is noteworthy that neuropathic pain, like other forms of chronic pain, significantly affects patients' social, economic and psychological well‐being in addition to their quality of life.^22^ Anxiety and depression are two psychological illnesses that are frequently linked to chronic neuropathic pain.^23^, ^24^ In general, chronic pain travels via nerve fibers to the spinal cord, the brain, the limbic and hypothalamic regions. In these domains, the spinal cord is crucial to the ascending pain pathway from the body to the brain because it triggers the release of neurotrophins and cytokines from activated neuronal cells, which are then part of the pain signaling cascade.^25^, ^26^ Pharmacological treatment is often accompanied by severe side effects and unsatisfactory outcomes^27^ due to the high cost of drugs, and treating chronic pain has been a major challenge for physicians and research groups.

In China, acupuncture has been used for more than 3000 years as a safe and effective means of treating acute and chronic pain in both humans and animals.^28^, ^29^ EA therapies are now widely utilized around the world, with acupuncture needles used to administer various amounts of electric stimulation to acupoints. For acute diseases, one acupuncture treatment session can typically result in a significant and fast therapeutic benefit. Repeated EA sessions may result in more reliable and comprehensive treatment regimens for chronic illnesses. Our current findings show that, compared to single or double episodes, the nociceptive thresholds of SNI mice increased during multiple EA sessions. Similarly, another study found that EA therapy at the Weizhong (BL40) and Huantiao (GB30) acupoints for 1 week alleviated NPP in CCI rats.^30^ It has been noted that several neurotransmitters and neuromodulators in the peripheral and central nervous systems, including norepinephrine, glutamate, g‐amino‐butyric acid (GABA), opioid peptides, serotonin and adenosine are involved in pain management by electroacupuncture.^31^ Sowa et al., 2010 reported that CD73 inhibits nociceptive circuits by hydrolyzing ATP to ADO, in turn sufficiently attenuating nociception.^32^ PAP and CD73 are the main ectonucleotidases that generate adenosine in nociceptive circuits.^33^ These enzymes produce an inhibitory adenosinergic signal from pulsatile or continuous nucleotide release. They revealed that the ectonucleotidase CD73 modulates nociception by hydrolyzing AMP to adenosine in nociceptive circuits, making adenosine a promising molecular target for chronic pain therapy. Furthermore, our findings also show that CD73 is a significant contributor to the genesis of adenosine at the level of SCDH. Adenosine acts through four receptors (Adora1, A2A, A2B and Adora3)^34^ that have been identified and cloned in various tissues.^35^ Among these, A1AR and A2A AR have antinociceptive properties^36^; however, A1 AR‐ and A2A‐mediated analgesia has been accompanied by severe cardiovascular side effects; hence, the use of A1AR and A2AAR agonists for therapeutic approaches has stalled.^37^, ^38^ A3AR is expressed in inflammatory, endothelial, neuronal, and glial cells in nervous tissue.^39^ The antinociceptive effects of A3AR agonists have been tested in several experimental NPP models to mitigate pain.^11^, ^40^, ^41^

In this study, we used an SNI model to induce NPP and explored the impacts of repeated EA on nociceptive pain behaviors and Adora3 expression in SCDH mice. SNI instigated substantial mechanical allodynia and thermal hypersensitivity and the nociceptive thresholds of the afflicted paw were significantly reduced and were lowest on Day 8 following SNI surgery, consistent with previous observations.^42^, ^43^ PWT (ipsilateral) and TWL showed that mice receiving repeated EA treatments experienced a significant cumulative reduction in pain due to increased adenosine levels, compared to non‐EA treated mice. These results highlight the importance of repeated EA treatment in achieving sustained adenosine‐related analgesia and the potential involvement of adenosine in mediating antinociception. Similar findings were presented in a study showing that the pain threshold was greater after successive sessions of EA over a long duration (weeks) than immediately after the session.^7^ Thus the cumulative analgesic effect following repeated EA observed in our study is consistent^44^, ^45^, ^46^, ^47^ and comparable with previous findings.

Correspondingly, after repeated EA sessions, the expression levels of Adora3 and CD73 mRNA and proteins were upregulated after every EA session and were significantly upregulated in the SNI + EA groups, with moderate downregulation of ADA mRNA and protein expression. Repeated EA treatment activated adenosine signaling after SNI in a neuropathic pain model. Previously, a possible mechanism for eliminating neuropathic pain through repeated EA sessions by suppressing ERK intracellular and p38 MAPK signaling in the hippocampus was postulated.^48^ Likewise, in another study it was found that repeated EA intervention had a cumulative analgesic effect on injury‐induced NPP reactions, resulting in synaptic remodeling of hippocampal neurons and increased calcium/calmodulin‐dependent protein kinase II expression in the hippocampal CA3 region.^47^ It has also been reported that the application of electroacupuncture stimulus at the acupoint ST36 diminishes neuropathic pain through the mediation of the spinal opioid receptor and the alpha2‐ and beta‐adrenoceptors.^49^ Double immunofluorescence labelling also verified the strong colocalization of spinal Adora 3 with Neun in the SCDH after repeated EA sessions. Fluorescent intensity was significantly lower in SNI mice and this was reversed by repeated electroacupuncture treatments. Similarly, adenosine A3 receptor agonists had antinociceptive effects in experimental NPP models^50^ All these findings strongly support the cumulative analgesic effects of repeated EA treatment. Peripheral nerve damage may play a role in the onset and persistence of NPP.^51^ Systemic administration or spinal application of adenosine and its exogenous analogues has antinociceptive effects on various pain models.^36^

The results of this investigation offer a new understanding of the molecular processes by which repeated EA sessions mediate antinociception and alleviate neuropathic pain via the Adora3 signaling pathway. Our findings demonstrated the significance of ADO in the longer‐lasting reduction of neuropathic pain induced by repeated EA sessions. In addition, the finding that the antinociceptive effect of EA is mediated via activation of the A3 AR located in the spinal cord is also quite intriguing. As a result, drugs that therapeutically interfere with A3 AR or adenosine metabolism may increase the clinical benefits of EA and provide a new route for the development of pain medications in the future. Although the study shows encouraging results, extending these findings to human patients requires additional validation through clinical studies. Furthermore, to fully confirm repeated EA efficacy, the study should be expanded to include validation in descending pain inhibitory systems. Moreover, this study exclusively used male mice, prompting the need for a similar investigation in female animals to evaluate the effectiveness of repeated EA in both genders.

## 5 CONCLUSIONS

In conclusion, repeated EA sessions ameliorate pain‐related behaviors (PWT and TWL) in SNI mice and subsequently upregulate spinal Adora 3 and CD73 mRNA and protein expression, and the application of repeated EA sessions in the SNI model upregulates the fluorescence intensity of Adora 3 to mitigate NPP.

## AUTHOR CONTRIBUTIONS

FAK, DM and DY participated in the design of the study. FAK, LH, and ZQ performed the experiments. GP and FAK acquired the data. FAK, LH, and MMA analyzed the data. FAK and DY drafted the manuscript. DM revised the manuscript. All authors read and approved the final manuscript.

## FUNDING INFORMATION

This research was funded by the National Nature Science Foundation of China, grant number 32172930.

## CONFLICT OF INTEREST STATEMENT

The authors declare no competing financial interests.

## ETHICS STATEMENT

Ethical approval was granted (HZAU‐20240105001) for the use and care of experimental animals from the institutional animal care and use committee of Huazhong Agricultural University by observing the regulations for the administration of affairs concerning experimental animals of China.
